# RASA: Robust Alternative Splicing Analysis for Human Transcriptome Arrays

**DOI:** 10.1038/srep11917

**Published:** 2015-07-06

**Authors:** Junhee Seok, Weihong Xu, Ronald W. Davis, Wenzhong Xiao

**Affiliations:** 1 School of Electrical Engineering, Korea University, Seoul 136-701, Korea; 2 Stanford Genome Technology Center, Palo Alto, CA 94304, USA; 3 Massachusetts General Hospital and Shriners Hospital for Children, Boston, MA 02114, USA

## Abstract

Human transcriptome arrays (HTA) have recently been developed for high-throughput alternative splicing analysis by measuring signals not only from exons but also from exon-exon junctions. Effective use of these rich signals requires the development of computational methods for better gene and alternative splicing analyses. In this work, we introduce a computational method, Robust Alternative Splicing Analysis (RASA), for the analysis of the new transcriptome arrays by effective integration of the exon and junction signals. To increase robustness, RASA calculates the expression of each gene by selecting exons classified as not alternatively spliced. It then identifies alternatively spliced exons that are supported by both exon and junction signals to reduce the false positives. Finally, it detects additional alternative splicing candidates that are supported by only exon signals because the signals from the corresponding junctions are not well detected. RASA was demonstrated with Affymetrix HTAs and its performance was evaluated with mRNA-Seq and RT-PCR. The validation rate is 52.4%, which is a 60% increase when compared with previous methods that do not use selected exons for gene expression calculation and junction signals for splicing detection. These results suggest that RASA significantly improves alternative splicing analyses on HTA platforms.

Alternative splicing of mRNA is a major mechanism that generates diverse mRNA transcript isoforms from a single gene, and subsequently differentiates proteins to have varying binding properties, intercellular localizations, enzymatic activities, and expression regulations^1^,^2^. Alternative splicing has been observed across tissue types, between distinct responses to external stimuli and among different developmental stages of mammalian stem cells^3^,^4^,^5^. Recent genome-wide studies reported that more than 90% of genes undergo alternative splicing^6^,^7^. More importantly, splicing variants are found in many human diseases such as Alzheimer’s disease, cystic fibrosis, heritable diseases, and cancers^8^,^9^,^10^. These variants are one of the major causes of the diseases^11^, and are targeted as biomarkers in disease diagnosis, prognosis and treatment^12^. Therefore, it is important to survey genome-wide splicing events in human health and diseases.

The massive parallel sequencing on mRNA (mRNA-Seq) has been actively used to study alternative splicing in a high-throughput manner^6^,^7^. Combined with newly developed computational methods, mRNA-Seq analyses enable us to quantify the abundance of transcript isoforms and discover novel isoforms^13^,^14^,^15^. At the same time, as a complimentary of mRNA-Seq, especially to analyze well-annotated isoforms, human transcriptome arrays (HTAs) have been developed^16^,^17^,^18^,^19^,^20^. With a high density of oligonucleotide probes, these arrays cover the whole exonic regions of the human genome as well as junction regions between two adjacent exons. For example, the recently released Affymetrix HTA 2.0^21^ covers ~560 k exons and ~340 k exon-exon junctions of the human genome. The HTAs have relatively low cost (about $250 per sample in the US including reagents) and short processing time, which makes the HTAs as a good complementary tool of mRNA-Seq for clinical studies that often require several hundreds or thousands of samples^20^. While a number of such studies are underway using HTA platforms^22^, computational challenges remain to effectively utilize the rich exon and junction signals in the data.

There have been several computational methods to detect alternative splicing using exon and junction signals. Analysis of splicing by isoform reciprocity (ASPIRE) algorithm detects splicing events by comparing inclusion and exclusion ratios calculated from corresponding junction probes^18^,^19^. Splicing index (SI) algorithm can be extended to accumulated splicing index (ASI), to score alternative splicing events by summing up the normalized expression fold changes of all exons and junctions related to a target event^16^. It is also possible to use junction probes solely to detect alternative splicing events with a probe affinity model^17^.

Despite these efforts, improving the detection accuracy is still a major challenge in alternative splicing analyses of microarray data^23^. For example, the conventional calculation of gene expression uses signals from all exons of a gene, regardless of whether an exon is alternatively spliced. This decreases the sensitivity of the subsequent alternative splicing analyses. In addition, while exon-exon junction probes are available on HTA platforms, and conceptually junction signals are more specific than exon signals to the alternative splicing events, analyzing junction data is more challenging than analyzing exon data. This is because junction probes are usually designed by tiling across each junction region (about 30 bp) leaving little room for optimization, so the probes are usually very similar in their sequences and likely either perform well or fail together. In contrast, exon probes are selected and optimized from the whole exon region, which is about 120 bp on average for a human gene.

To address these challenges, here we propose a new algorithm, named as Robust Alternative Splicing Analysis (RASA), to reduce the false positives of detecting alternative splicing events on HTA and similar exon-junction arrays. In order to reduce the biases caused by alternative splicing events on the calculation of gene expression, the method first calculates the expression index of each gene with only selected exons of the gene that are not differentially spliced between the sample groups of the study. It then detects alternatively spliced exons by requiring not only significant signal from the exon regions but also additional supporting evidence from the corresponding junctions, to reduce false positive detections. Further, since in a typical study, not all the junctions are detected with reliable signals, at the final step, the algorithm reports those additional candidates of alternatively spliced exons where the corresponding junctions are not measured reliably on the arrays. These candidates can then be reviewed and selected for further verification, by RT-PCR, for example.

We tested RASA using samples of human liver and muscle. Three replicated samples for each tissue were hybridized on Affymetrix HTA, which has been used in previous studies^24^. The detection performance of the proposed method was assessed by an independent mRNA-Seq data set^7^. In addition, nine candidates were selected for RT-PCR and were validated. The algorithm was also applied to data of a custom-designed HTA platform^20^ between liver and muscle with four replicates for each condition. Finally we applied RASA on an additional dataset of T cells and monocytes isolated from ten healthy individuals. These results show that RASA substantially improve the performance of alternative splicing analysis of data of HTA platforms.

## Methods and Materials

### Summary of the HTA data analyses

Following standard protocols suggested by the manufacturer, three replicates of human liver and muscle tissue samples were hybridized on Affymetrix HTA 1.0, previously high-density human exon junction array, HJAY^24^. Raw probe intensities were summarized into gene, exon and junction expression indices using a standard procedure of GC background correction^25^, median-scaling normalization^26^, and median-polish summarization^27^. Alternative splicing of exons and junctions were statistically tested using MIDAS^28^ and MADS^23^ methods. Normalized expression fold changes were computed as fold changes of exon and junction expression divided by gene expression, as like splicing indices^16^. The presence of exons and junctions per sample were tested by DABG^29^. The presence in an experiment condition was represented by a group DABG p-value, a geometric mean of sample DABG p-values in the condition. The custom-designed HTA data of liver and muscle, four replicates for each, was obtained from GSE26072^20^, and processed in a similar way. A diagram of the overall procedure of the proposed algorithm is shown in Supplementary Figure S1.

### Putatively constitutive exon selection

Putatively constitutive exons of a gene are defined as exons that are not alternatively spliced across conditions, and the remaining exons of the gene are putatively alternative. The proposed method starts with the full set of exons and excludes putatively alternative exons until no more exons can be removed. For a simple comparison of two conditions, first, exons absent in any condition are marked as putatively alternative (group DABG p-value > 0.01 in this work). Among remaining exons, one is marked as an outlier if its expression fold change falls outside of [*m* − *ds*, *m* + *ds*], where *m* and *s* are the mean and standard deviation of exon expression fold changes respectively, and *d* is a pre-defined sensitivity parameter. In this work, the default value of *d* was chosen to be 2, which allowed small changes within two-fold of the standard deviation. *m* and *s* are calculated again with non-outlier exons and outliers are updated. This process is repeated until outliers converge into a fixed set, which becomes to be additional putatively alternative exons. The gene expression is calculated from probes of the remaining putatively constitutive exons.

### Alternatively spliced exon detection with supporting junctions

Several metrics, such as MIDAS, MADS, normalized expression fold changes (or Splicing Indices) and DABG, are available to detect alternatively spliced exons^16^,^23^,^28^,^29^. Here, we describe a simple case using only MIDAS p-values, but other metrics are equally applicable. First, significant exons are selected by a pre-defined threshold *p*. To compensate the systematic difference between exons and junctions, significant junctions are selected with an adjusted threshold *p’*, which is given as *p’* = *Q*^−1^_junc_(*Q*_exon_(*p*)), where *Q*_exon_ and *Q*_junc_ represent quantile functions of exon and junction p-values respectively and *Q*^−1^ is the inverse function of *Q*. If a significant exon has at least one significant junction over- or less-expressed together, the exon is considered as a confident candidate of alternative splicing. If an exclusion junction is significantly spliced in the opposite direction, it can also support the splicing of exons.

### Detection of alternatively spliced exons with unreliable junctions

For a significantly spliced exon, its junctions are considered to be unreliable if their expression changes are much smaller than the adjacent exon. As an upper bound, each significant exon is compared with its best junction that has the largest expression change in the same direction with the exon expression change among all junctions. If no junction changes in the same direction, the expression change of the best junction is truncated to 0. The difference of expression fold changes of an exon and its best junction is calculated in a log scale. The extremeness of fold change difference is measured against a null distribution that is estimated by alternatively spliced exons detected with supporting junctions. This null distribution can be considered as a true distribution of an exon and its best junction working properly. Given a pre-defined quantile threshold *q*, a decision boundary is set as the value corresponding to the top *q* quantile of the null distribution. If the fold change difference of an exon is larger than this decision boundary, the exon is determined to have unreliable junctions. If these exons are significantly spliced by themselves, they are considered as confident candidate of alternative splicing without junction supports. In this work, the default value of *q* was chosen to be 0.05, which could be considered as a 5% significance level in the empirical test for unreliable junctions.

## Results and Discussion

### Step 1: gene expression calculation using putatively constitutive exons

In the analysis of microarray data, accurately calculating gene expression indices is essential because an alternative splicing event between two groups of samples in a study is detected by comparing the changes in expression indices of an exon or a junction relative to the changes in the expression of the corresponding gene^16^,^18^,^19^. When gene expression is calculated from probes of all the exons of a gene, it inevitably includes changes caused by exons differentially spliced in the data of the study^29^. Consequently, changes are artificially introduced into the calculated gene expression indices because of these alternatively spliced exons, which results in the loss of sensitivity in the detection of differential splicing.

In order to minimize this bias, methods were proposed previously to estimate gene expression index using only annotated constitutive exons, which are defined as exons that are included in all transcript isoforms of a gene in a given gene annotation^18^. These constitutive exons can be identified from the annotations of RNA transcripts in RefSeq, Ensembl, and other publicly available databases^30^. However, a substantial percentage of the genes do not have sufficient number of constitutive exons for robust estimation of gene expression. As an example, in the transcript annotations used for a custom-designed HTA design^20^, 40% of genes have no constitutive exons at all.

Instead, we used an unsupervised approach in RASA to iteratively select putatively constitutive exons from the data, and then use them to calculate gene expression more robustly. Putatively constitutive exons are selected as ones that, in all the conditions within a specific study, are uniformly present and show similar expression changes with the overall gene expression changes across these conditions. The rest of the exons are considered to be putatively or conditionally alternative, and excluded from gene expression calculations. Note that here the putatively alternative exons do not necessarily imply alternatively spliced, since some of them can be called because they do not appear to exist in any of the conditions of the study or their signal varies too much in the data to be included in the calculation of gene expression.

The proposed approach is also distinct from an approach using “core exons” as described in Risueno *et al.*^31^, where the “core exons” are defined as common exons in the protein-coding transcripts that cover more than 60% of the gene. The use of core exons can be considered as an intermediate approach between the calculations using all exons and using annotated constitutive exons. This approach has two potential problems. One problem is that the gene expression calculation is affected by alternative spliced exons that are currently annotated as core exons. The other problem is that some genes might have too few or no such core exons, especially when a comprehensive gene annotation is used. In contrast, the proposed method detects “putatively constitutive exons” from the data of the study. It can exclude from gene expression calculation any exons which show strong alternative splicing signals in the data. Moreover, it can include in the gene expression analysis those exons that are not shown to be alternatively spliced in the data of the study, regardless of whether the exons are annotated as core (or constitutive) exons in the database, since many of the exons are not alternatively spliced under the specific conditions of the study even though they are annotated in the database as alternatively spliced under some other conditions.

**Figure 1:**
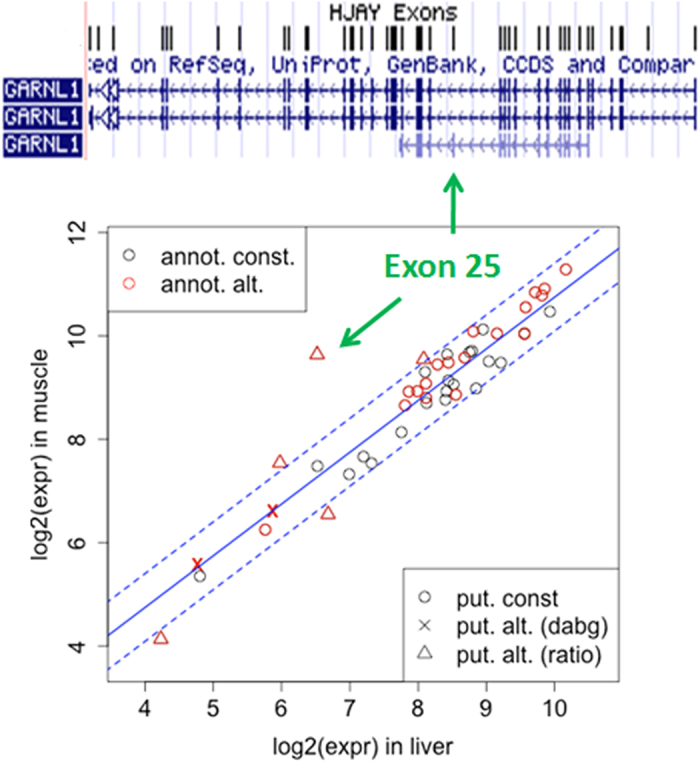
Putatively constitutive exon selection in GARNL1. At the top, the gene structure of GARNL1 from UCSC Known Gene is shown with HTA exon probe sets. At the bottom, logged exon expression indices of liver and muscle are plotted. Black and red colors represent annotated constitutive and alternative respectively as in the design database of the HTA. The blue bold line represents an average expression ratio over all putative constitutive exons identified by RASA, and blue dashed lines show upper and lower boundaries set in the algorithm to call putative alternative exons. A putatively constitutive exon identified by RASA is marked with a circle. A putatively alternative exon identified by RASA is either categorized as absent in the sample (marked with a cross) or as significantly deviated from the average expression ratio of the putative constitutive exons (triangle). Exon 25 of the gene is later identified as an alternatively spliced exon between the tissues.

Figure 1 shows an example of putatively constitutive exons between liver and muscle samples in the HTA data. The blue bold line has slope 1 and an interceptor whose value is the log ratio of expression indices between liver and muscle. Among the 46 exons of gene GARNL1, 21 are annotated constitutive and 25 annotated alternative exons, according to the design database of HTA. From the array data of liver and muscle, RASA selected 39 exons as putatively constitutive exons including 21 annotated constitute exons and 18 annotated alternative exons in the design database. In the figure, the performance of these 18 annotated alternative exons aligned well with the 21 annotated constitutive exons, indicating that they were not alternatively spliced between liver and muscle. The rest 7 exons were identified by RASA as putatively alternative exons. Among these, two were absent in both liver and muscle tissues, four were on the detection boundary of the alternative splicing analysis, and one exon (exon 25 of GARNL1, chr14: 35, 239, 119 ~ 35, 239, 259) showed a significantly different pattern of expression than all the other exons of GARNL1.

### Step 2: alternative splicing detection with supporting junction information

After gene expression indices are calculated, RASA assesses the significance of alternative splicing of each exon and each junction between two (or more) groups of interest in the study. This is done by one or a combination of statistical tests such as MIDAS^28^ and MADS^23^. While MIDAS and MADS have been developed for statistical tests of alternative splicing in exon arrays which do not have junction information, these methods can be directly applied to test alternative splicing of junctions.

In RASA, exons with significant differential splicing signals are identified, and all the junctions connected with each of these exons are examined. Only those exons supported by at least one junction with significant splice signal are selected as confident candidates of alternative splicing. By requiring significant splicing of both exons and their associated junctions, the approach reduces false positive detections. Note that one significant connecting junction is sufficient to support the finding that the exon is differentially spliced. This is because, while in the design database many junctions can connect to an exon, we expect only significant changes for the junction(s) connecting the exon to an isoform whose level significantly changed between the groups of interest in the study. For other junctions connecting the exon to isoforms with little changes, significant changes of junction signals are not expected.

Since junction probes are designed from the junction regions (~30 bp) which are much shorter than the exon regions (~120 bp), the numbers of junction probes (typically 4 ~ 8) are often fewer than those of exon probes (typically 8 ~ 10), and these junction probes are often less optimized than the exon probes. Because of the systematic difference in the number and performance between exon and junction probes, the estimated significances of alternative splicing signals of exons and junctions have different distributions. In order to compensate for this, the p-value distribution of the junctions is empirically adjusted according to the distribution of the exons p-values. In the testing study, significantly spliced exons were detected with MIDAS p-value threshold of 0.01, which corresponded to the top 5.9% of exon p-values, and significant junctions were detected with an adjusted p-value cutoff of 0.007, corresponding to the same top 5.9% of junction p-values.

### Step 3: identification of unreliable junctions and listing additional candidates of alternative splicing

At this step, RASA detects additional candidate exons of differential splicing that are missed in the previous step, because signals from their potential supporting junctions fail to be measured reliably. This is achieved by comparing fold changes of the expression indices of the exon and the junctions connecting to it. Since in alternatively splicing, different junctions connect the same end of an exon to different adjacent exons, which result in different gene isoforms, a junction is in general more specific to the isoform where it resides comparing with the exon that can be shared by the multiple isoforms created. As the result, if junction signals are measured properly, junctions have larger or at least equal fold changes in expression indices comparing to the corresponding exons. Therefore, if an exon is detected to be significantly spliced by itself but all of its junctions have extremely smaller fold changes than the exon, the signals of the junctions are likely unreliable. The difference in expression changes between an exon and its junctions can be tested over a null distribution estimated using those alternatively spliced exons detected with supporting junctions in the previous step (Step 2). This null distribution is expected to reflect the true distribution from alternatively spliced exons and their junctions behaving properly.

**Figure 2:**
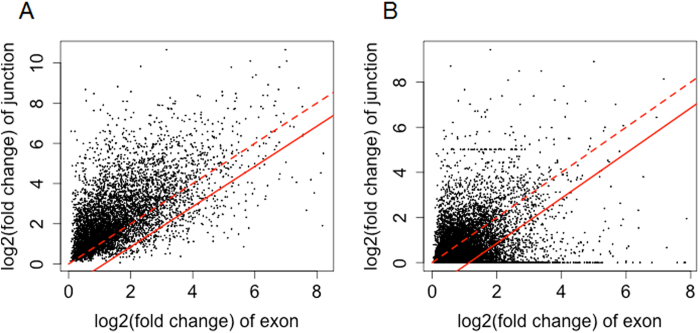
Identification of exons with unreliable junctions. Normalized fold changes of exons and their corresponding junctions for **(A)** significant exons supported by significant junctions, and **(B)** significant exons without significant junctions. Dashed red lines present the equal fold changes of exons and junctions, and solid red lines present the cutoff threshold corresponding to 5% significance. Exons on the bottom-right side of the threshold line are considered to have significantly larger fold changes than their junctions.

Figure 2 shows how unreliable signals of junctions are detected between the liver and muscle data. First, a null distribution was estimated with significant exons having at least one significant junction. In the null distribution, top 5% exons, which have the largest fold change difference between the exon and its junctions, are selected, and a cutoff threshold of the fold change difference is then determined for the detection of unreliable junction signal (Fig. 2A). Among the exons that were detected to be significant by themselves but not supported by significant junctions, only 15% of exons satisfied the cutoff threshold (Fig. 2B) and were additionally selected as splicing candidates. A weak correlation in Fig. 2A is expected because a spliced junction tends to have a larger or equal fold change comparing with the corresponding exon, but the fold change difference can vary between different pairs of exons and junctions. Even though there are some exceptions due to noise, the spliced exons have significant junctions with larger or similar fold changes (Fig. 2A). This clear pattern is not observed in exons without significant junctions (Fig. 2B).

### Performance benchmarks

**Figure 3:**
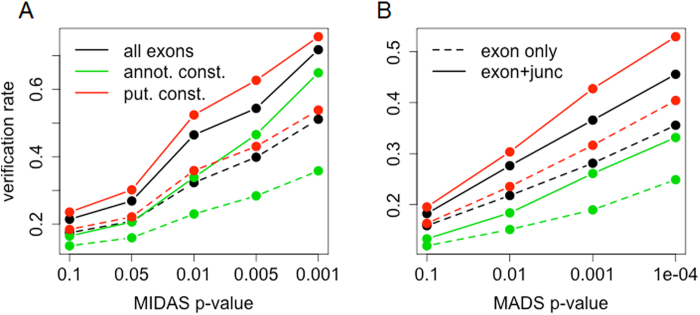
Performance comparison. Comparisons of the performance of various methods to detect alternatively spliced exons. Verification rates are shown along with **(A)** MIDAS p-values and **(B)** MADS p-values. The methods calculating gene expression with all exons, annotated constitutive exons, and putatively constitutive exons are noted with black, green and red lines respectively. The detection results with and without junction supports are represented with bold and dashed lines respectively. The performance of RASA is plotted with red bold lines.

The proposed RASA algorithm was tested using data of human liver and muscle tissue samples hybridized on Affymetrix HTA 1.0 arrays. The detected candidates were further validated with an independent mRNA-Seq data set on the same tissues^7^. The overall result clearly shows that the combination of putatively constitutive exon selection and alternative splicing detection with junction supports significantly helps the identification of true positive candidates of alternative spliced exons (Table 1). In the array data, our method achieved a 52.4% validation rate, which was a 60% increase when comparing with the approach that calculate gene expression using all exons and detect alternative splicing with only exon signals, which have been widely used in previous studies^28^,^29^. In addition, over the wide range of significance criteria in both MIDAS and MADS tests, the proposed RASA algorithm performed better than the other approaches (Fig. 3). The verification rates were calculated from 200 ~ 1,700 verifiable candidates from the mRNA-Seq data, which are large enough to ensure that the benchmark results are robust. A similar outperformance of the proposed method was observed from the analyses of custom-designed HTA data (Supplementary Figure S2). Importantly, the performance of the method was robust to the changing parameters of the algorithm, e.g. sensitivity parameter *d* and quantile threshold *q*. Comparing to the 52.4% validation rate with the default parameters (*d* = 2 and *q* = 0.05), the validating rate remained within 47% ~ 57% over a wide range of these parameters (Supplementary Figure S3).

**Figure 4:**
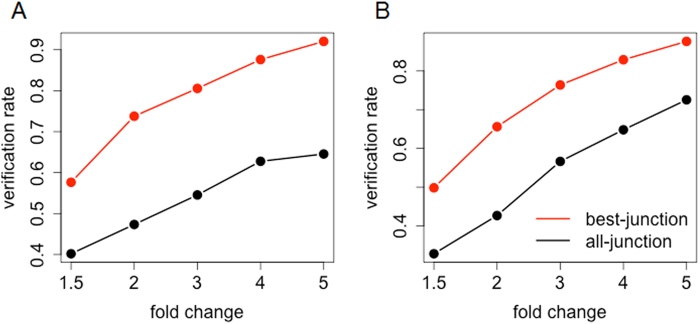
Performance comparison with all-junction approaches. Performance comparison of **(A)** ASPIRE and **(B)** ASI with the proposed method. Red lines represent validation rates of the proposed method using a best-junction approach, and black lines represent the other two methods using an all-junction approach. RASA detected candidates first with a given fold change threshold (x-axis), the same number of top candidates by ASPIRE and ASI were compared, and the verification rates by mRNA-Seq data were presented (y-axis).

The performance of RASA was also compared with that of ASPIRE^18^,^19^ and ASI^16^, two previously introduced methods utilizing junction information in alternative splicing analyses. Since both ASPIRE and ASI use the fold changes of normalized exon and junction expression as their primary measures of alternative splicing, our method was also demonstrated with the normalized expression fold changes. For a given fold change threshold, our method first detects confident candidates, and the same number of top candidates from ASPIRE and ASI were compared. As shown in Fig. 4, the proposed method shows better validation rates than both ASPIRE and ASI. With a two-fold cutoff, our method had 65 ~ 75% validation rates which was a >50% increase from the other two approaches. A similar result was obtained from the analysis of the custom-designed HTA data (Supplementary Figure S4). One potential reason for this observation is that, while our method follows a “best-junction” approach that requires only one good junction to support an exon with significant alternative splicing signal, the other two methods follow an “all-junction” approach that incorporates information from all the junctions associated with the exon. Since both supporting and non-supporting junctions are utilized together in the all-junction approach, it is likely difficult to identify alternative splicing signals clearly.

To determine an exon as a confident candidate of alternative splicing, RASA requires strong signals from the exon and one of its junctions as a supporting junction. Alternatively, an exon can be detected with only strong junction signals by not requiring strong signals from the exon itself. For example, MADS+^32^ follows this approach by just requiring one of inclusion probe sets (exons or junctions that are included in an alternative splicing event) and one of exclusion probe sets (exons or junctions that are skipped). MADS+ allows two junctions to support a splicing event while RASA requires one exon and one junction to have strong signals. Because of short exon-exon junction regions, the design of junction probes are often difficult to be optimized, which makes junction probes less reliable than exon probes. Therefore, the approach not requiring strong exon signals might produce more false positive detections. Comparing the MADS+ approach with RASA in similar settings with the benchmark tests, RASA shows better verification rates over a wide range of MADS p-value criteria (Supplementary Figure S5).

**Figure 5:**
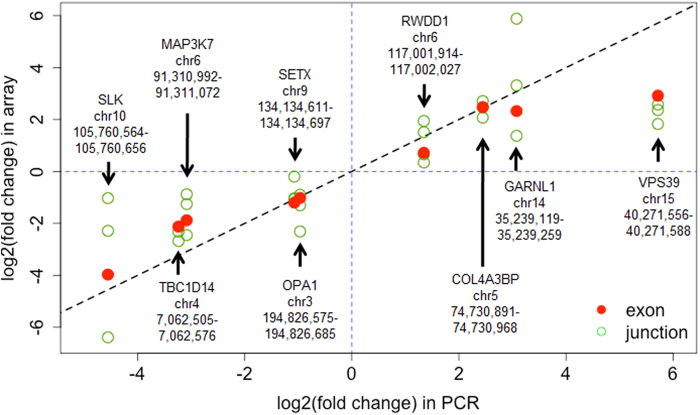
RT-PCR verification. Comparison of HTA and RT-PCR results of nine selected alternative splicing candidates identified by RASA. Shown are fold changes of the expression levels of exon and junctions measured between liver and muscle. A positive logged fold change indicates over-expression in muscle over liver, and vice versa. Red and green circles represent exons and junctions respectively.

Some candidates from the HTA analysis were not determinable by the mRNA-Seq analysis because they were not covered by enough sequencing reads. In order to further test these candidates, we performed additional verification experiment using RT-PCR. We selected nine such candidates based on signal strength and simplicity of gene structures. As shown in Fig. 5, the PCR results confirmed all these nine candidates with good agreements of expression fold changes.

In addition, RASA was tested using the data of human T-cell and monocyte samples. T-cell and monocyte samples from each of 10 healthy individuals were hybridized on a custom designed HTAs (Supplementary Methods). The analysis found 708 alternatively spliced exons (p-value < 0.01), among which 44.1% were verified by independent mRNA-Seq data of the same tissues. This is a significant improvement from the analysis without the use of junction signals and putatively constitutive exons, of which verification rate was only 29.7%. Similar to the results of the previous two datasets, in this dataset RASA also showed better verification rates over a wide range of significance criteria (Supplementary Figure S6). As an example, the NIN (Ninein) gene was shown in Supplementary Figure S7, where an exon of the gene is detected as alternative spliced by the HTA analysis and verified by the mRNA-Seq data. These results support the ability of RASA for larger data sets with high variations, such as in a clinical setting.

The reliability of the RASA analysis is expected to be dependent on the within and between group variations of the samples. In the analysis of liver and muscle samples, RASA worked well even with three replicates by achieving a 52.4% validation rate because these were technical replicates with relatively small variations. In the analysis of T-cells and monocytes, larger variation between samples of the same cell type from the 10 different individuals might explain the somewhat lower validation rate (44.1%). In a real clinical application with patient samples, the sample variations are expected to be even larger than between healthy individuals. In such a case, more samples will likely be required to have enough powers for the analysis.

## Conclusion

In this work, we describe RASA for the alternative splicing analyses of HTA platforms. The algorithm is based on two properties of junction signals: (1) if an exon is alternatively spliced, at least one of the junctions linked to the exon should be also alternatively spliced, and (2) if an exon is alternatively spliced, at least one of its junctions is expected to have the larger or at least equal expression fold changes comparing with that of the exon. Based on the first property, we determine an exon as a confident candidate of alternative splicing when the exon itself is significantly spliced and at least one of its junctions is also significantly spliced. Based on the second property, we detect exons with unreliable junctions if all junctions of the exon have much lower expression fold changes than that of the exon. The distribution of fold changes of spliced exons and reliable junctions are empirically estimated from the confident candidates of the previous step. This distribution is used as a null distribution and if all the junctions of an exon have extremely lower fold changes that fall below a certain percentile of the null distribution, we consider the exon to have unreliable junction signals. We include these exons as additional candidates for alternative splicing if they are significantly spliced by themselves without supporting junctions.

RASA was tested between liver and muscle samples hybridized on the Affymetrix HTA arrays, and verified with independent mRNA-Seq data. The rate of validation was 52.4%, a 60% increase from the previous approaches. Additional analyses of custom-designed HTA data sets showed similar results. The overall result shows that alternative splicing detection with RASA can significantly improve the performance of the analysis. This method is being further tested in the analysis of HTA data of a clinical study to investigate splicing of traumatic patients. The implementation of RASA can be found at http://igenomed.stanford.edu/~junhee/RASA/.

## Additional Information

**How to cite this article**: Seok, J. *et al.* RASA: Robust Alternative Splicing Analysis for Human Transcriptome Arrays. *Sci. Rep.* **5**, 11917; doi: 10.1038/srep11917 (2015).

## Supplementary Material

Supplementary Information

## Figures and Tables

**Table 1:** Summary of performance benchmarks.

**Exon Selection**	**Alternative Splicing**	**Number of Determinable Candidates**	**Number of Verified Candidates**	**Rate**
Constitutive exons	Exon only	618	142	23.0%
All exons	Exon only	1,738	561	32.4%
Putatively constitutive exons	Exon only	1,506	540	35.9%
Putatively constitutive exons	With junctions	695	364	52.4%

The performance summarization of various methods in the detection of alternatively spliced exons with MIDAS p-value < 0.01 and DABG p-value < 0.01. It shows the number of determinable candidates, the number of verified splicing events by the mRNA-Seq analyses, and the verification rate.

## References

[b1] Black D. L. Mechanisms of alternative pre-messenger RNA splicing. Annu Rev Biochem 72, 291–336 (2003). 12626338 10.1146/annurev.biochem.72.121801.161720

[b2] Stamm S. *et al.* Function of alternative splicing. Gene 344, 1–20 (2005). 15656968 10.1016/j.gene.2004.10.022

[b3] Ip J. Y. *et al.* Global analysis of alternative splicing during T-cell activation. RNA 13, 563–572 (2007). 17307815 10.1261/rna.457207 PMC1831861

[b4] Pritsker M., Doniger T. T., Kramer L. C., Westcot S. E. & Lemischka I. R. Diversification of stem cell molecular repertoire by alternative splicing. Proc Natl Acad Sci U S A 102, 14290–14295 (2005). 16183747 10.1073/pnas.0502132102 PMC1242282

[b5] Xu Q., Modrek B. & Lee C. Genome-wide detection of tissue-specific alternative splicing in the human transcriptome. Nucleic Acids Res 30, 3754–3766 (2002). 12202761 10.1093/nar/gkf492 PMC137414

[b6] Pan Q., Shai O., Lee L. J., Frey B. J. & Blencowe B. J. Deep surveying of alternative splicing complexity in the human transcriptome by high-throughput sequencing. Nat Genet 40, 1413–1415 (2008). 18978789 10.1038/ng.259

[b7] Wang E. T. *et al.* Alternative isoform regulation in human tissue transcriptomes. Nature 456, 470–476 (2008). 18978772 10.1038/nature07509 PMC2593745

[b8] Faustino N. A. & Cooper T. A. Pre-mRNA splicing and human disease. Genes & development 17, 419–437 (2003). 12600935 10.1101/gad.1048803

[b9] Garcia-Blanco M. A., Baraniak A. P. & Lasda E. L. Alternative splicing in disease and therapy. Nat Biotechnol 22, 535–546 (2004). 15122293 10.1038/nbt964

[b10] Kim E., Goren A. & Ast G. Insights into the connection between cancer and alternative splicing. Trends in genetics : TIG 24, 7–10 (2008). 18054115 10.1016/j.tig.2007.10.001

[b11] Lopez-Bigas N., Audit B., Ouzounis C., Parra G. & Guigo R. Are splicing mutations the most frequent cause of hereditary disease? FEBS letters 579, 1900–1903 (2005). 15792793 10.1016/j.febslet.2005.02.047

[b12] Brinkman B. M. Splice variants as cancer biomarkers. Clin Biochem 37, 584–594 (2004). 15234240 10.1016/j.clinbiochem.2004.05.015

[b13] Jiang H. & Wong W. H. Statistical inferences for isoform expression in RNA-Seq. Bioinformatics 25, 1026–1032 (2009). 19244387 10.1093/bioinformatics/btp113 PMC2666817

[b14] Kim D. *et al.* TopHat2: accurate alignment of transcriptomes in the presence of insertions, deletions and gene fusions. Genome Biol 14, R36 (2013). 23618408 10.1186/gb-2013-14-4-r36 PMC4053844

[b15] Trapnell C. *et al.* Transcript assembly and quantification by RNA-Seq reveals unannotated transcripts and isoform switching during cell differentiation. Nat Biotechnol 28, 511–515 (2010). 20436464 10.1038/nbt.1621 PMC3146043

[b16] Clark T. A., Sugnet C. W. & Ares M. Jr. Genomewide analysis of mRNA processing in yeast using splicing-specific microarrays. Science 296, 907–910 (2002). 11988574 10.1126/science.1069415

[b17] Johnson J. M. *et al.* Genome-wide survey of human alternative pre-mRNA splicing with exon junction microarrays. Science 302, 2141–2144 (2003). 14684825 10.1126/science.1090100

[b18] Licatalosi D. D. *et al.* HITS-CLIP yields genome-wide insights into brain alternative RNA processing. Nature 456, 464–469 (2008). 18978773 10.1038/nature07488 PMC2597294

[b19] Ule J. *et al.* CLIP identifies Nova-regulated RNA networks in the brain. Science 302, 1212–1215 (2003). 14615540 10.1126/science.1090095

[b20] Xu W. *et al.* Human transcriptome array for high-throughput clinical studies. Proc Natl Acad Sci U S A 108, 3707–3712 (2011). 21317363 10.1073/pnas.1019753108 PMC3048146

[b21] Affymetrix, *GeneChip Human Transcriptome Array 2.0 Data Sheet.* (2013) Available at http://www.affymetrix.com/support/technical/datasheets/hta_array_2_0_datasheet.pdf (Accessed: 15th December 2014).

[b22] Wang P. *et al.* The STAT3-binding long noncoding RNA lnc-DC controls human dendritic cell differentiation. Science 344, 310–313 (2014). 24744378 10.1126/science.1251456

[b23] Xing Y. *et al.* MADS: a new and improved method for analysis of differential alternative splicing by exon-tiling microarrays. RNA 14, 1470–1479 (2008). 18566192 10.1261/rna.1070208 PMC2491471

[b24] Lin L. *et al.* Using high-density exon arrays to profile gene expression in closely related species. Nucleic Acids Res 37, e90 (2009). 19474342 10.1093/nar/gkp420 PMC2709591

[b25] Affymetrix, *Exon array background correction.* (2005) Available at http://www.affymetrix.com/support/technical/whitepapers/exon_background_correction_whitepaper.pdf (Accessed: 15th December 2014).

[b26] Kapur K., Xing Y., Ouyang Z. & Wong W. H. Exon arrays provide accurate assessments of gene expression. Genome Biol 8, R82 (2007). 17504534 10.1186/gb-2007-8-5-r82 PMC1929160

[b27] Irizarry R. A. *et al.* Exploration, normalization, and summaries of high density oligonucleotide array probe level data. Biostatistics 4, 249–264 (2003). 12925520 10.1093/biostatistics/4.2.249

[b28] Affymetrix, *Alternative transcript analysis methods for exon arrays.* (2005) Available at http://www.affymetrix.com/support/technical/whitepapers/exon_alt_transcript_analysis_whitepaper.pdf (Accessed: 15th December 2014).

[b29] Clark T. A. *et al.* Discovery of tissue-specific exons using comprehensive human exon microarrays. Genome Biol 8, R64 (2007). 17456239 10.1186/gb-2007-8-4-r64 PMC1896007

[b30] Seok J., Xu W., Jiang H., Davis R. W. & Xiao W. Knowledge-based reconstruction of mRNA transcripts with short sequencing reads for transcriptome research. PloS one 7, e31440 (2012). 22312447 10.1371/journal.pone.0031440 PMC3270033

[b31] Risueno A. *et al.* A robust estimation of exon expression to identify alternative spliced genes applied to human tissues and cancer samples. BMC Genomics 15, 879 (2014). 25297679 10.1186/1471-2164-15-879 PMC4298068

[b32] Shen S., Warzecha C. C., Carstens R. P. & Xing Y. MADS+: discovery of differential splicing events from Affymetrix exon junction array data. Bioinformatics 26, 268–269 (2010). 19933160 10.1093/bioinformatics/btp643 PMC2804303

