# Supplementary material for: RASA: Robust Alternative Splicing Analysis for Human Transcriptome Arrays
Source: Sci Rep. 2015 Jul 6;5:11917. doi: 10.1038/srep11917 (PMC4491729; doi:10.1038/srep11917)
Supplement: Supplementary Information [file srep11917-s1.pdf]

Supplementary Materials

RASA: Robust Alternative Splicing Analysis for Human Transcriptome Arrays

Junhee Seok[1*], Weihong Xu[2], Ronald W. Davis[2], Wenzhong Xiao[2,3*]

[1]School of Electrical Engineering, Korea University, Seoul 136-701, Korea.

[2]Stanford Genome Technology Center, Palo Alto, CA 94304, USA

[3]Massachusetts General Hospital and Shriners Hospital for Children, Boston, MA 02114, USA.

[*]Corresponding Author

Emails

JS: jseok14@korea.ac.kr

WXiao: wenzhong.xiao@mgh.harvard.edu

Supplementary Methods

Verification of HTA results by mRNA-Seq data: High-throughput RNA sequencing data of human liver and muscle tissues was obtained from GSE12946[1]. Sequencing reads were mapped over the exonic regions, allowing up to two nucleotide mismatches by SeqMap[2]. An exon expression index was calculated as the number of reads per kilobase per million reads (RPKM) using only uniquely mapped reads[3]. A gene expression index was computed in a similar way using reads aggregated from all exons that belong to the gene.

To verify HTA analyses, one robust set of alternatively spliced exons and another robust set of non-spliced exons were determined from the mRNA-Seq data, using p-values from statistical tests for relative over-expression of exons (p), the number of mapped reads (n), and fold changes of exon expression normalized to gene expression (f). The over-expression of exons relative to gene expression was tested by Fisher's exact tests[4]. For a simple comparison between two conditions (C1 and C2), an exon is determined to be *strictly over-expressed* in C1 if $p < 0.001$, $n \geq 20$ and $f > 2$, or *strictly not over-expressed* in C1 if $p > 0.5$, n in the C2 ≥ 20, and the exon has the larger normalized expression in C2 than in C1. For both conditions, four sets (strictly over-expressed in C1, strictly not over-expressed in C1, strictly over-expressed in C2, and strictly not over-expressed in C2) were determined, and exons that were not in any of four sets were classified to be *not determinable*. If an exon detected to be over-expressed in one condition by a microarray analysis is also determined to be strictly over-expressed in the same condition in mRNA-Seq data, it is counted as a true positive (TP). If it is determined to be strictly not over-expressed in the same condition, it is counted as a false positive (FP). The verification rate is defined as (# of TPs)/(# of TPs + # of FPs).

Several mRNA-Seq tools for alternative splicing analysis, such as Cufflinks[5], Casper[6], and DiffSplice[7], are based on the estimation of isoform-level expression. An objective for RASA is to identify specific exons as alternative splicing candidates that can be verified using RT-PCR. Since it is not easy to confidently extract exon-level alternative splicing events from the isoform-level differential expression, we employed a conservative method to check the alternative

splicing of each exon based on the number of mapped reads, expression fold changes, and statistical tests using Fisher's exact tests, which have been used in many previous works[1,4,8,9].

ASPIRE and ASI method: The proposed method was compared with the analysis of splicing by isoform reciprocity (ASPIRE) algorithm[10,11], and the accumulated splicing index (ASI)[12]. For the alternative splicing score of an exon, ASPIRE calculates an inclusion ratio using both inclusion and exclusion junctions of the exon. ASI calculates the sum of splicing indices of an exon and all of its junctions as the score. Both ASPIRE and ASI detect top several exons ranked by their scores as confident candidates of alternative splicing. While ASI was tested over all exons, ASPIRE was tested over a subset of exons whose exclusion junctions are available because ASPIRE requires exclusion junctions in its calculation.

RT-PCR validation: Primer pairs were designed using Primer3 package, and manually reviewed to ensure differentiation between PCR products of different transcript isoforms. RT-PCR experiments were performed following a standard protocol on the 7900HT Fast Real-Time PCR System (Life Technology, Inc). Cycle numbers (Ct) to reach a pre-determined threshold in exponential increment phase were recorded. For a candidate exon, RT-PCR experiments were repeated three times, and the average Ct was used to estimate the expression of the candidate exon. Gene expression was estimated from the expression of adjacent constitutive exons of the candidate alternative exon. To verify alternative splicing, the odd ratio of splicing index was computed as the ratio between the fold change of exon expression and the fold change of gene expression.

Additional test data of human T-cell and monocyte samples: RASA was tested using human T-cell and monocyte samples. 10 biological replicates of each cell type were hybridized on a custom-designed HTA[13]. These samples were obtained from 10 healthy individuals as a part of the Glue Grant study, *Inflammation and the Host Response to Injury* (www.gluegrant.org). By sequencing the pool of the 10 replicates for each cell type using an Illumina Hi-Seq machine, 90 million and 98 million reads were obtained for the T-cell and monocyte samples, respectively. The mRNA-Seq data were processed by Cufflink[5] with the default option to consider ambiguous read mapping, and the expression indices of genes and exons were calculated accordingly. The

detected splicing events by RASA from the HTA data were compare with the mRNA-Seq data from the same samples.

Supplementary References

1 Wang, E. T. *et al.* Alternative isoform regulation in human tissue transcriptomes. *Nature* **456**, 470-476 (2008).

2 Jiang, H. & Wong, W. H. SeqMap: mapping massive amount of oligonucleotides to the genome. *Bioinformatics* **24**, 2395-2396 (2008).

3 Mortazavi, A., Williams, B. A., McCue, K., Schaeffer, L. & Wold, B. Mapping and quantifying mammalian transcriptomes by RNA-Seq. *Nat Methods* **5**, 621-628 (2008).

4 Bullard, J. H., Purdom, E., Hansen, K. D. & Dudoit, S. Evaluation of statistical methods for normalization and differential expression in mRNA-Seq experiments. *BMC Bioinformatics* **11**, 94 (2010).

5 Trapnell, C. *et al.* Transcript assembly and quantification by RNA-Seq reveals unannotated transcripts and isoform switching during cell differentiation. *Nat Biotechnol* **28**, 511-515 (2010).

6 Rossell, D., Stephan-Otto Attolini, C., Kroiss, M. & Stocker, A. Quantifying Alternative Splicing from Paired-End Rna-Sequencing Data. *The annals of applied statistics* **8**, 309-330 (2014).

7 Hu, Y. *et al.* DiffSplice: the genome-wide detection of differential splicing events with RNA-seq. *Nucleic Acids Res* **41**, e39 (2013).

8 Brooks, A. N. *et al.* Conservation of an RNA regulatory map between Drosophila and mammals. *Genome Res* **21**, 193-202 (2011).

9 Katz, Y., Wang, E. T., Airoldi, E. M. & Burge, C. B. Analysis and design of RNA sequencing experiments for identifying isoform regulation. *Nat Methods* **7**, 1009-1015 (2010).

10 Licatalosi, D. D. *et al.* HITS-CLIP yields genome-wide insights into brain alternative RNA processing. *Nature* **456**, 464-469 (2008).

11 Ule, J. *et al.* CLIP identifies Nova-regulated RNA networks in the brain. *Science* **302**, 1212-1215 (2003).

12 Clark, T. A., Sugnet, C. W. & Ares, M., Jr. Genomewide analysis of mRNA processing in yeast using splicing-specific microarrays. *Science* **296**, 907-910 (2002).

13 Xu, W. *et al.* Human transcriptome array for high-throughput clinical studies. *Proc Natl Acad Sci U S A* **108**, 3707-3712 (2011).

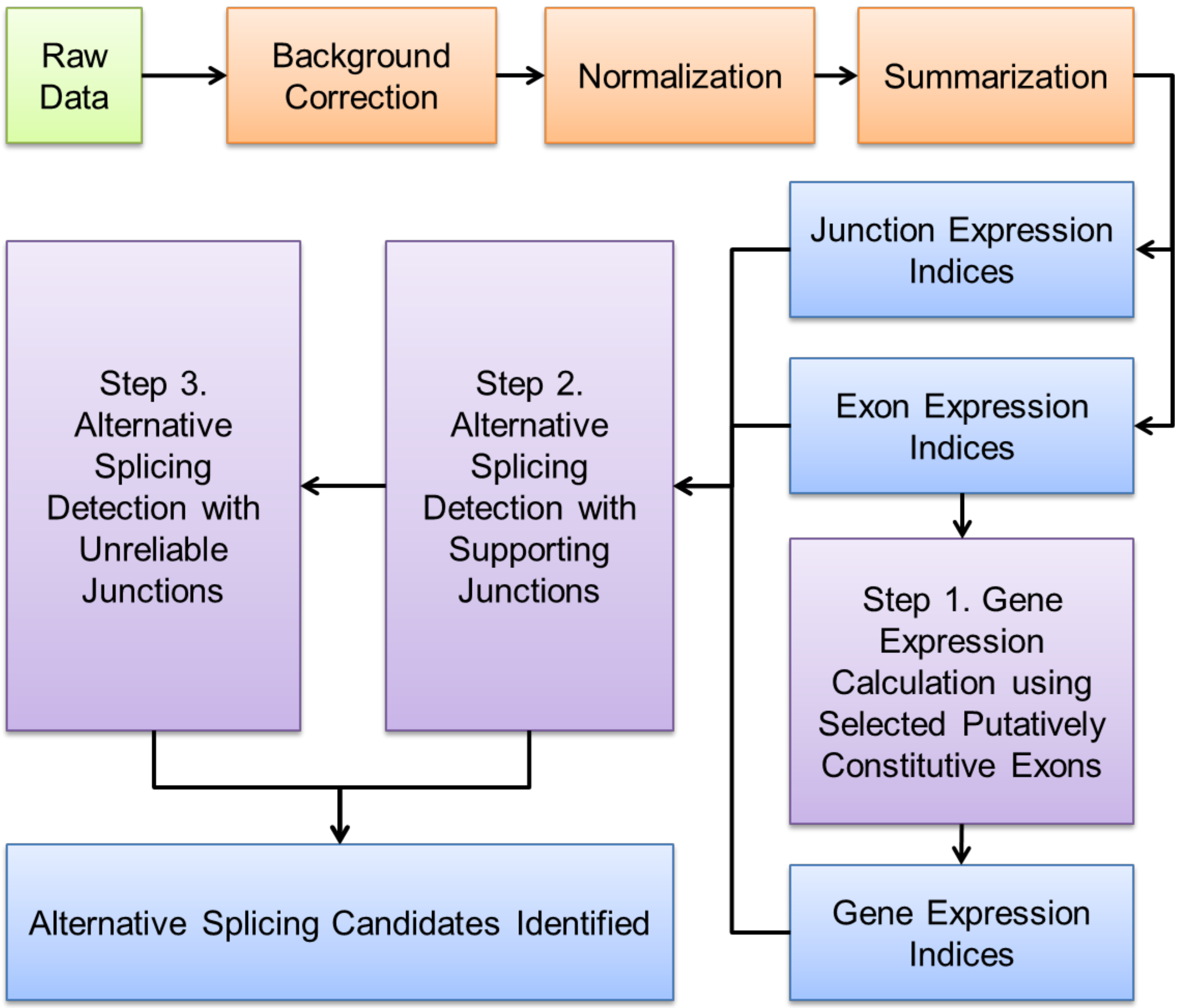

Figure S1. Diagram of the overall procedure of RASA. Orange boxes are preprocessing steps of the microarray data. Blue boxes are major outputs of the program. Purple boxes are the major steps of the proposed algorithm for alternative splicing analysis.

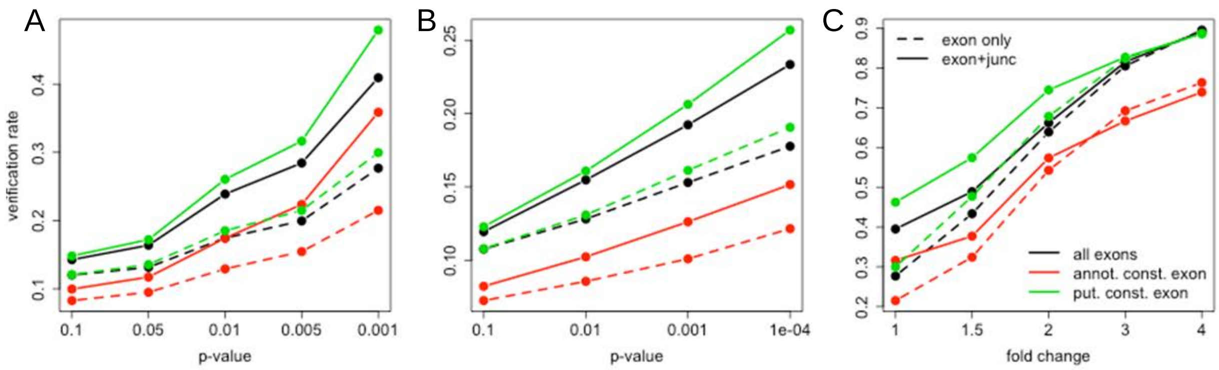

Figure S2. Performance benchmarks on the custom-designed HTA data. Verification rates are also shown with microarray detection parameters of **(A)** MIDAS p-values, **(B)** MADS p-values, and **(C)** exon expression fold changes relative to gene expression fold changes. The methods calculating gene expression with all exons, annotated constitutive exons, and putatively constitutive exons are noted with black, red and green lines respectively. The detection results with and without junction supports are represented with bold and dashed lines respectively. The RASA results are presented with green and solid lines.

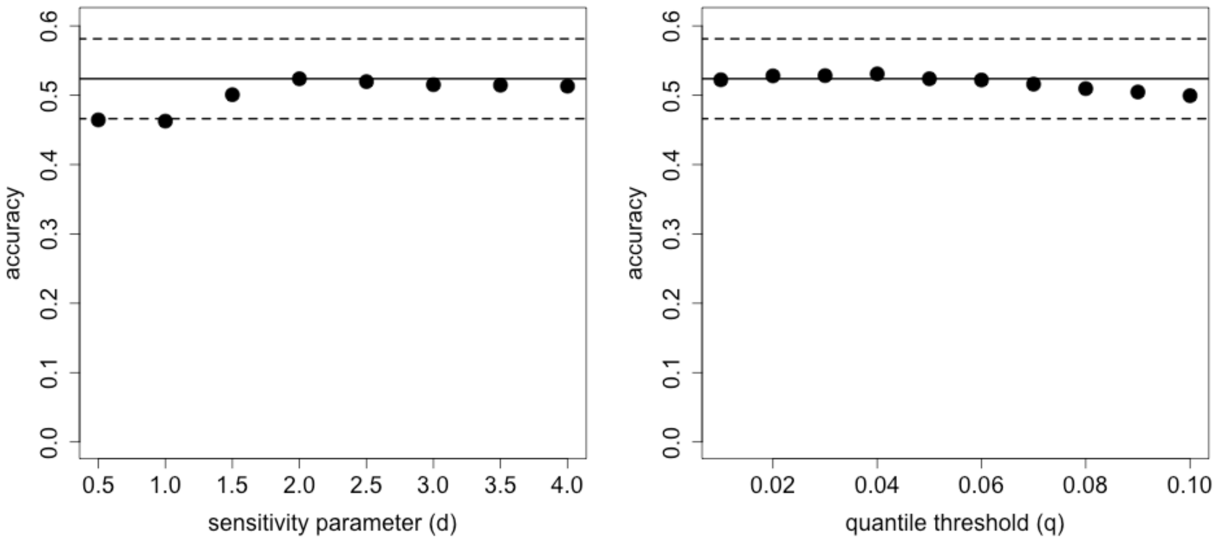

Figure S3. Performance changes over a range of parameters. The performance of RASA over the changes of **(A)** sensitivity parameter *d* and **(B)** quantile threshold *q*. The bold black lines represent the detection accuracy with the default parameters (d=2 and q=0.05). The dashed lines represent the boundary of 10% changes from the accuracy with default parameters.

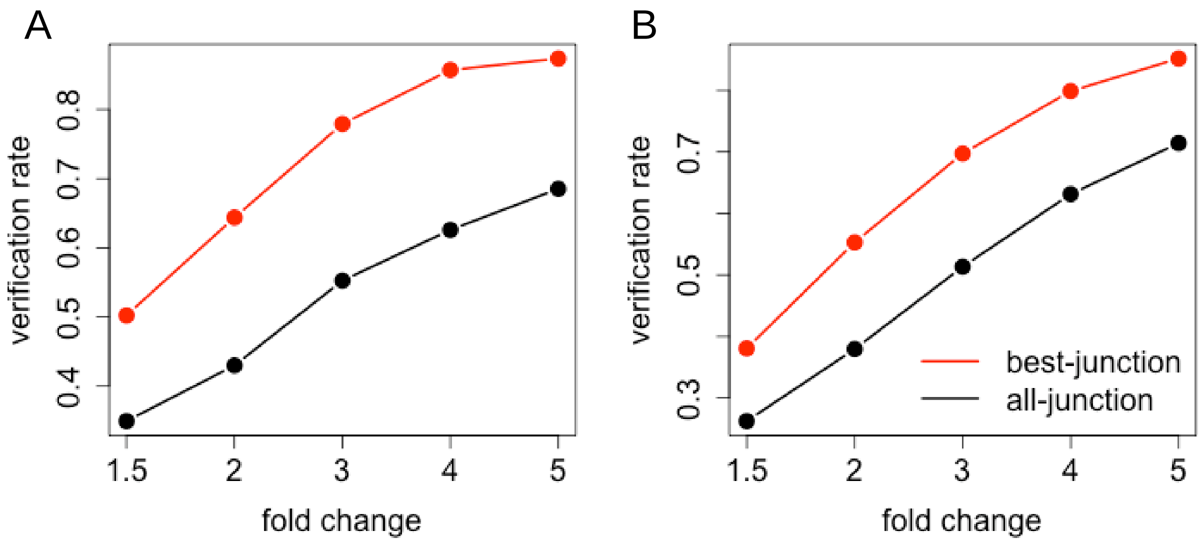

Figure S4. Performance comparison with all-junction approaches on the custome-designed HTA data. Performance comparison of **(A)** ASPIRE and **(B)** ASI with the proposed method using the custom-designed HTA data. Red lines represent validation rates of the proposed method of a best-junction approach, and black lines represent the other methods of all-junction approaches. The x-axis shows fold change criterions used for the proposed method.

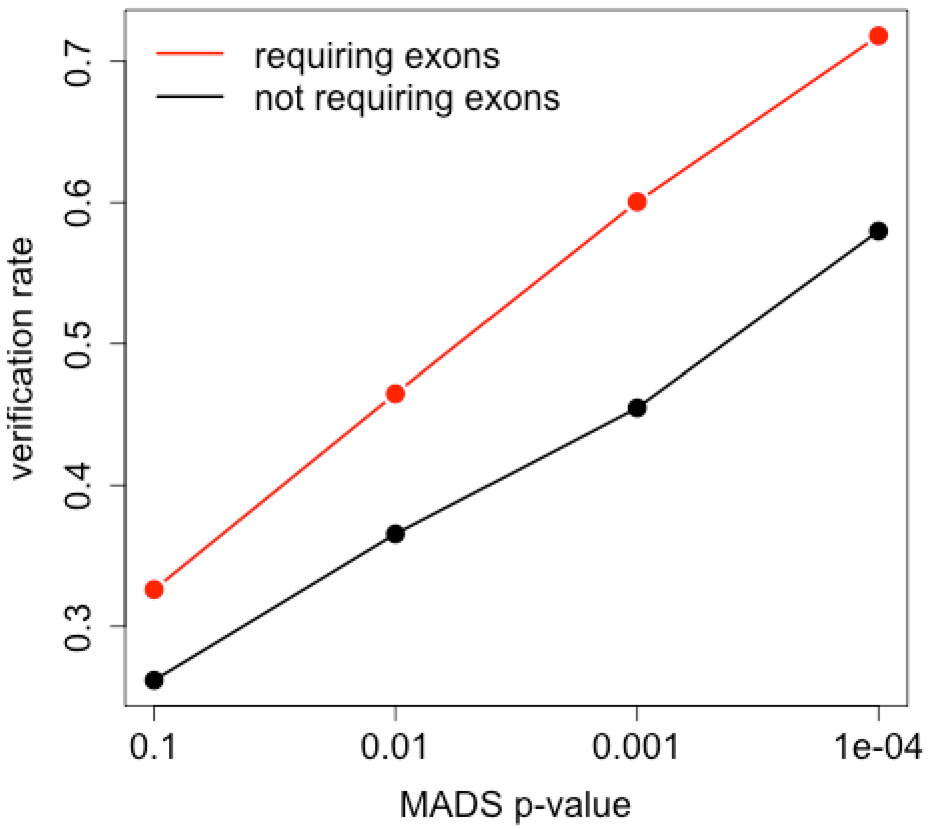

Figure S5. Performance comparison with MADS+. Comparison between the approach of RASA that requires strong signals from exons (red), and the approach of MADS+ that do not requiring strong exon signals. The detections by RASA and MADS+ were performed over the same set of exons.

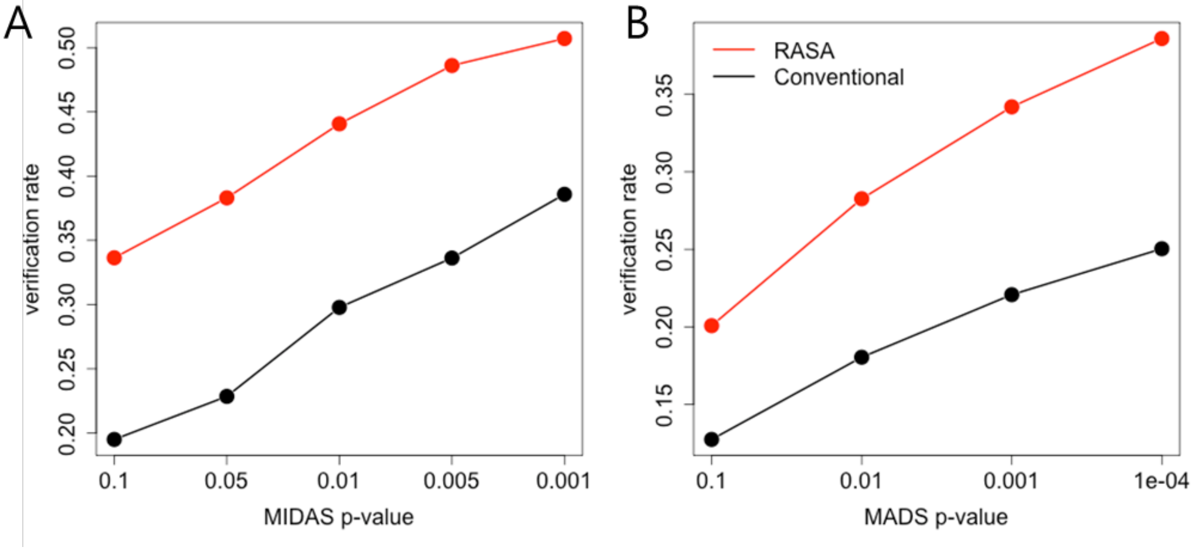

Figure S6. Performance comparison with T-cell and monocyte samples. Comparisons of the performance of RASA (red) and conventional method (black) to detect alternatively spliced exons between human T-cells and monocytes. Verification rates are shown along with **(A)** MIDAS p-values and **(B)** MADS p-values. RASA used putative constitutive exon selection for gene expression calculation and both exon and junction signals for the detection of alternative splicing as proposed. The conventional method used all exons for gene expression calculation and only exon signals for the detection of alternative splicing.

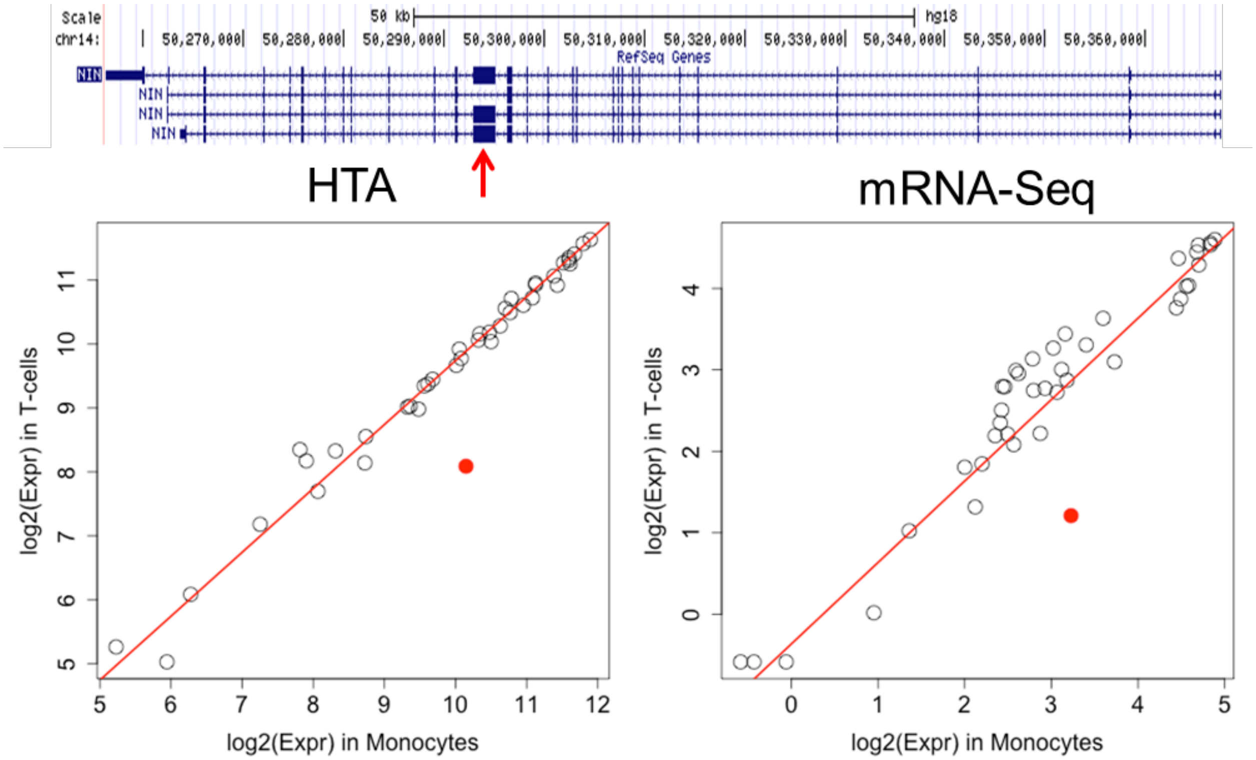

Figure S7. Alternative Splicing in NIN gene. An exon (chr14: 50,292,960-50,295,098) of Ninein (NIN) gene is shown as a confident candidate of alternative splicing between human T-cells and monocytes (red arrow in the gene annotation; red dots in the plots). The plots show the expression of exons of NIN from the HTA and mRNA-Seq data (black circles). Red lines represent the averages of fold changes of exons.
